# A conservation and biophysics guided stochastic approach to refining docked multimeric proteins

**DOI:** 10.1186/1472-6807-13-S1-S7

**Published:** 2013-11-08

**Authors:** Bahar Akbal-Delibas, Nurit Haspel

**Affiliations:** 1 Department of Computer Science, University of Massachusetts Boston, Boston, MA 02125, USA

## Abstract

**Background:**

We introduce a protein docking refinement method that accepts complexes consisting of any number of monomeric units. The method uses a scoring function based on a tight coupling between evolutionary conservation, geometry and physico-chemical interactions. Understanding the role of protein complexes in the basic biology of organisms heavily relies on the detection of protein complexes and their structures. Different computational docking methods are developed for this purpose, however, these methods are often not accurate and their results need to be further refined to improve the geometry and the energy of the resulting complexes. Also, despite the fact that complexes in nature often have more than two monomers, most docking methods focus on dimers since the computational complexity increases exponentially due to the addition of monomeric units.

**Results:**

Our results show that the refinement scheme can efficiently handle complexes with more than two monomers by biasing the results towards complexes with native interactions, filtering out false positive results. Our refined complexes have better IRMSDs with respect to the known complexes and lower energies than those initial docked structures.

**Conclusions:**

Evolutionary conservation information allows us to bias our results towards possible functional interfaces, and the probabilistic selection scheme helps us to escape local energy minima. We aim to incorporate our refinement method in a larger framework which also enables docking of multimeric complexes given only monomeric structures.

## Background

### Protein binding and docking

Proteins often associate with other proteins to create complexes that function as a biological unit. These complexes play a central role in nearly every cellular process [1]. Since the structure and function of proteins are closely related, detection of protein complexes and their structures helps us understand their role in various important biological processes.

Despite the advance in experimental structure detection methods, elucidating the three-dimensional arrangement of protein complexes is still a very challenging process. Computational methods have become very useful in complementing and helping experimental structure detection methods. Computational docking methods try to predict the way two or more proteins bind. They are typically made of two stages: The search stage uses structural and geometric techniques to detect native-like configurations of the complex, and the ranking stage uses a scoring function made of physico-chemical and geometric filters to estimate the binding affinity and rank computed structures according to energetic criteria. These functions typically focus on electrostatic, Van der Waals, and solvent interactions, similarity to experimental structures, or agreement with other experimental data [2-9].

### Multimeric docking

In nature many proteins interact to generate multimers containing more than two monomeric units, but most docking and refinement methods only focus on dimeric structures due to the possible exponential increase in the already large search space, posed by the addition of monomers. Due to the additional increase in complexity, in the case of multimeric docking it is especially important to carefully select the search and ranking methods. Only a small number methods exist for docking more than two monomers. These methods attempt to make the search for the correct docking configuration tractable by focusing on symmetric complexes [10] or by extending pairwise solutions via combinatorially assembling monomers incrementally, using greedy heuristics to cut down the search space such as selecting only a subset of the complexes of size *k *and pass them to the next stage as candidates to search for a complex of size *k *+ 1, or generating pairwise docking results and expanding them using a minimum spanning tree [11,12].

### Docking refinement

The results generated by computational docking methods are expected to be low-energy structures that are similar to the native complex structures. However, computational docking methods are not complete. The energetic difference between the native structure and other non-native complexes may be small and the scoring function used by docking methods is often not sensitive enough to detect it. Additionally, the correct binding site is not always known experimentally and docking methods may miss the correct binding site completely. As a result, low-energy structures produced by docking programs often disagree with NMR data [13]. Recent CAPRI (Critical Assessment of PRedicted Interactions) rounds show an important observation: even the most accurate methods predict only about 50% of the targets [2]. A survey of various scoring functions showed that although some components in several scoring functions have meaningful individual components, none of these functions could predict the binding affinity reliably [14]. Therefore, the results of computational docking methods need to be further refined in order to obtain native-like structures. Usage of refinement methods on protein complexes is not limited to computational docking methods; structures obtained by experimental methods can also be refined. Docking algorithms often produce a large number of putative complexes, ranked according to some scoring function. Docking refinement methods refine and re-rank these complexes in order to produce improved structures with lower energy and better interface packing. The goal is to improve both the RMSD and the ranking of the solution closest to the native structure. Refinement methods are often based on a combination of geometric and energetic optimization. Existing methods include rigid body transformations with side chain flexibility [15,16], flexible fitting that accounts for the changes proteins undergo upon binding [17], normal-mode analysis [18,19], Molecular Dynamics (MD) [3,20], energy minimization [21], Monte Carlo (MC) [22], genetic algorithms [11] and more.

### Refinement and re-ranking using conservation and electrostatics

We recently developed a docking refinement method that uses a scoring function based on evolutionary conservation [23,24], in addition to the usual VdW energy term. It employs a novel Evolutionary Trace (ET)-based [25,26] conservation scoring function. Evolutionary Traces are based on the idea that residues on functional interfaces are important for correct binding, and are therefore more likely to be conserved. We showed a strong correlation between conservation scores and the correct binding geometry when tested on dimeric protein structures. Our method biases the search towards conformations which have those conserved amino acids positioned close to each other on the binding interface. The scoring function iteratively detects top-scoring transformations at each stage of the refinement and passes them to the next stage for further refinement. We use a greedy selection approach to avoid exponential growth of the number of candidate complexes and speed up the computation time. We showed that the method can significantly improve docking results and also help distinguishing badly docked complexes from near-native complexes.

More recently we extended our refinement method to multimeric protein structures [27]. Biasing the search towards functional interface greatly reduces the search space, which is especially important in the case of multimeric complexes. We also incorporated electrostatic interaction energy to improve the accuracy of our prediction and provide a greater diversity of the selected conformations. The search iteratively selects two monomers out of the complex, and they are refined with respect to each other. Out of the newly refined candidates, top ranking conformations with respect to energy are passed on to the next stage for further refinement. In that work we also introduced a new probabilistic search scheme, which allows a greater variety in the selection of complexes and enables the method to escape possible local minima. We showed that our refinement method significantly improved the geometry of the input complexes and achieved lower lRMSD with respect to the native complexes.

In the current work we introduce an improved scoring function which aims to eliminate the bias created by the conservation score towards large interfaces. As input, we use coarsely docked complexes resulting from a multimeric docking program, Multi-LZerD [11]. We tested our refinement method on a large dataset of both dimeric and multimeric complexes. In most cases, there are several results among the top ranking complexes with better lRMSD than the input structure. This shows the potential of our method to serve as an efficient tool to improve the geometry and interface packing of coarsely docked complexes.

## Methods

Our program takes as input a protein complex generated by any docking method. The refinement proceeds in cycles where each cycle seeks to improve the conformation of one unit (i.e., a chain or a list of chains) with respect to the other ones. For each input structure, we create 100 conformations using rigid-body rotations by a random angle within a predefined range around an arbitrary axis passing through the centroid of the unit. Each rotation results in a new conformation and these randomly generated conformations are first energy minimized for 200 steps using NAMD [28] to resolve local clashes without introducing drastic changes to the structure, then ranked using both a conservation scoring function and an electrostatic scoring function. After creating probability distributions based on conservation and electrostatic ranking, 10 conformations are selected according to the probabilistic selection scheme described below and provided as inputs for the following refinement cycle.

### Creating multimeric protein structures

The coarsely docked multimers used in this paper were produced using Multi-LZerD [11] without the refinement module. We selected coarsely docked complexes whose distance to the native complexes was between 1 and 6Å, to allow effective refinement and not attempt to refine incorrectly docked complexes whose RMSD from the native structure was too big to refine.

**Figure 1:**
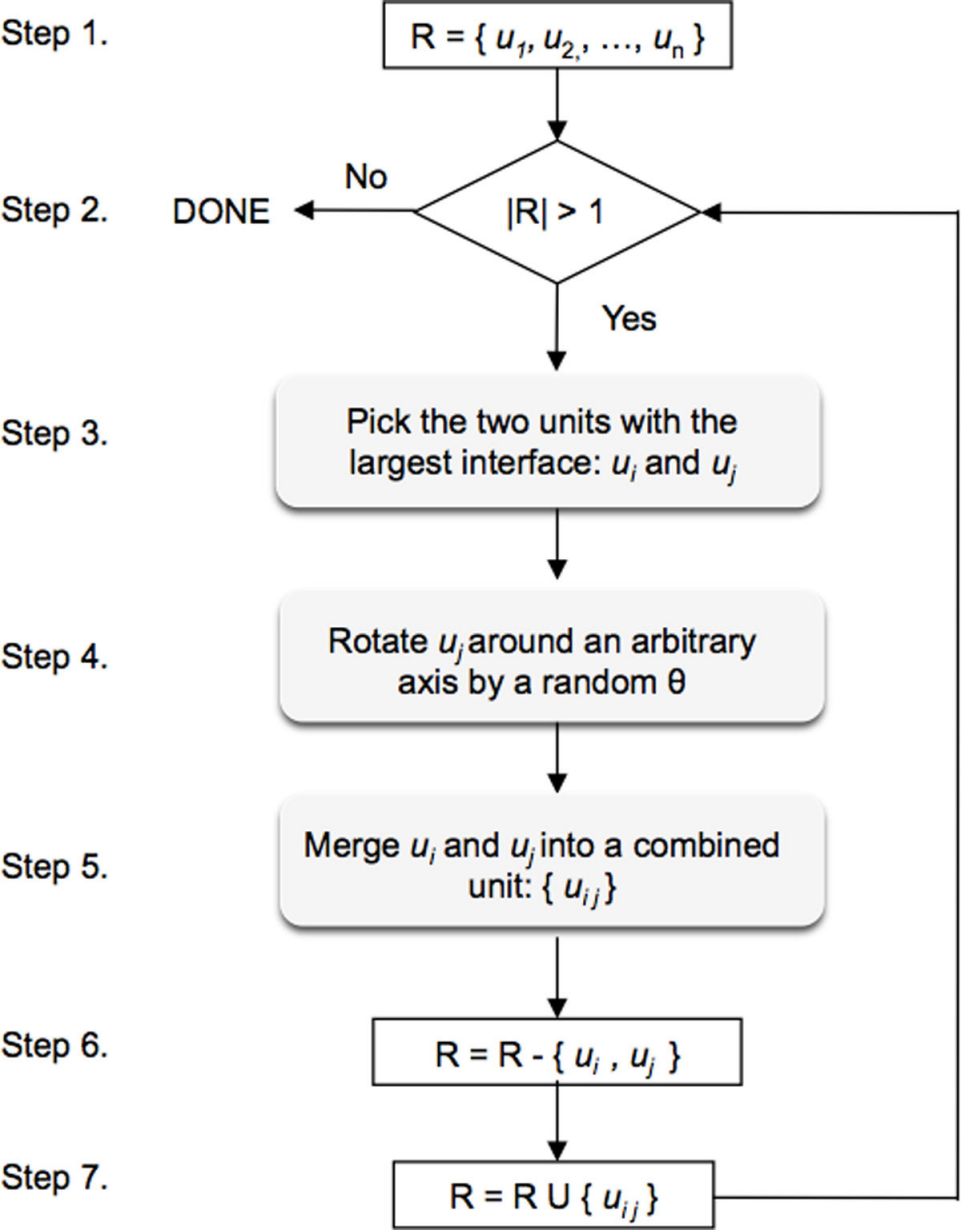
**Flowchart of creating an arbitrary conformation for a multimeric protein structure during the refinement process**.

We refine multimeric protein structures by creating conformations as described in the flowchart at Figure 1. We first create a set of units to refine, R (step 1). In the beginning each chain is considered a separate unit. We then do a pairwise interface comparison and pick the two units, *c_i _*and *c_j _*, in R that share the largest interface (step 3). Next, we rotate *c_j _*around an arbitrary axis passing through its centroid by a random angle between -5 and 5 degrees (step 4). Afterwards, we merge *c_i _*and *c_j _*into a *combined unit *(step 5), remove *c_i _*and *c_j _*from R (step 6) and add the new combined unit to R (step 7). This process repeats until R has a single combined unit that contains all the chains of the protein. By combining the units we achieve two important benefits: (i) we refine chains or chain lists in the order that leads to the largest interface, and (ii) we avoid impairing previously refined chains.

### Scoring function

The scoring function that we aim to optimize is computed for the set of interface atoms, which is defined, for each chain, as the atoms within at most 6 Å distance to an atom from an adjacent chain. In our previous work [23], we employed a scoring function consisting of the *Van der Waals *term taken from the AMBER ff03 force field [29] and the *conservation *term that we defined using ET scores of each interface residue.

For each interface atom, we defined the evolutionary conservation value, *c_i_*, as the relative importance of the residue that the atom belongs to. Relative importance of a residue is specified in the *coverage *column of the corresponding ET files for each protein chain. The coverage value ranges between 0 and 1, where low coverage implies evolutionary importance.

The conservation term of our interface scoring function was then defined as in Eq. (1), where *c_i _*and *c_j _*are the conservation values for the interface atom pair *i *and *j*. In this manner, each interface atom *i *on one unit and interface atom *j *on the other unit are considered in computing the conservation term.

(1) $$E_{conservation}=\underset{i,j}{\sum }c_{i}\;*\;c_{j}$$

By experiments on several protein complexes we have previously shown [23] that the proposed *conservation *term had strong correlations with least RMDS (lRMSD) values. Therefore, we defined the scoring function based on conservation (*E_TC_*) as in Eq. (2). Minimized Van der Waals term, *E_V dW _*was added to eliminate structures with clashing atoms.

(2) $$E_{TC}=E_{V\;dW}+E_{conservation}$$

Through experiments on different protein complexes, we showed in [27] that the scoring function defined in Eq. (2) proves useful also in refining multimeric protein complexes. On the other hand, we also identified that for some docked protein complexes the conservation-based scoring function does not show a strong correlation with lRMSD values. Yet the interface electrostatic energy, taken from the AMBER ff03 force field [29], is highly correlated with lRMSD values for those complexes. Therefore, we defined another scoring function based on electrostatic (*E_TE_*) as in Eq. (3). Similar to Eq. (2), *E_V dW _*is added to eliminate structures with steric clashes. Below we explain how to use these two scoring functions in combination.

(3) $$E_{TE}=E_{V\;dW}+E_{electrostatic}$$

### Probabilistic selection of conformations

We rank our refinement candidates using the above mentioned scoring function and select a subset of them as the refinement output. In our previous work [23], we ranked random conformations according to *E_TC _*values and selected the 10 top ranked conformations.

Deterministic selection increased the likelihood of false positives because we selected only top 1% (10 out of 2000) of conformations in a multi cycle refinement process. The scoring function rarely correlates perfectly with lRMSD values and is only a model of the "true" potential energy. Also, it increased our chances of getting trapped in a local minimum. In order to address this limitation, we employ a probabilistic selection approach detailed below, which we first introduced in [27].

The conformations are sorted in ascending order using the scoring functions defined in Eq. (2) and Eq. (3) and create two different probability distributions based on *E_TC _*and *E_TE _*values as in Table 1. We then randomly select 10 conformations according to the conservation score probability distribution and 10 conformations according to the electrostatic score probability distribution. The cumulative probability of selecting the top 10% conformations is about 70%, which allows lower energy conformations to be selected more often. In the future we will experiment with different selection probabilities and their effect on the results. We will also try to distinguish between complexes whose geometry correlates better with *E_TC _*and those that correlate better with *E_TE_*, as it appears that they represent different types of interface interactions.

**Table 1:** Probability distribution table.

Conformations	Relative Probability	Selection Probability (100 conf.)	Selection Probability (2000 conf)
Top 1%	1	0.2632	0.0132
Next 2%	0.5	0.1316	0.0066
Next 7%	0.1	0.0263	0.0013
Next 20%	0.02	0.0053	0.0003
Last 70%	0.01	0.0026	0.0001

After the conformations are sorted in ascending order according to *E_TC _*and *E_TE_*, their selection probability depending on the number of generated conformations (100 or 2000) is assigned as described above. The relative probability is with respect to a conformation in the top 1% to be selected.

### Test set

In order to test our multimeric refinement method, we used docked dimeric structures provided by Shehu et al. [24] with the following PDB IDs: 1BDJ, 1C1Y, 1CSE, 1DS6, 1OHZ, 1TX4 and 1WQ1. In addition to these dimers, we produced multimeric input structures by running the Multi-LZerD multimeric docking program without refinement [11] for protein complexes with the following PDB IDs: 1I3O, 1JYO, 1LOG, 1QGW, 1VCB, 1W88, 1WWW, 2BBK, 2PRG and 6RLX. Some of these proteins are trimers or tetramers that we used before as dimers only [23,30], while others are popular test cases [11].

For each input docked structure, the refinement is performed iteratively in 2 steps. In the first step, 100 random conformations are generated from the input structure as described in Section. These 100 conformations are ranked using the two scoring functions and 20 conformations are selected according to our selection function (10 according to *E_TC _*values and 10 according to *E_TE _*values). In the second step, 100 new random conformations are created for each of the 20 conformations produced in the first step. Then, these 2000 new conformations are ranked using the scoring functions and 20 conformations are selected and output as refined candidate complexes.

## Results and discussion

**Figure 2:**
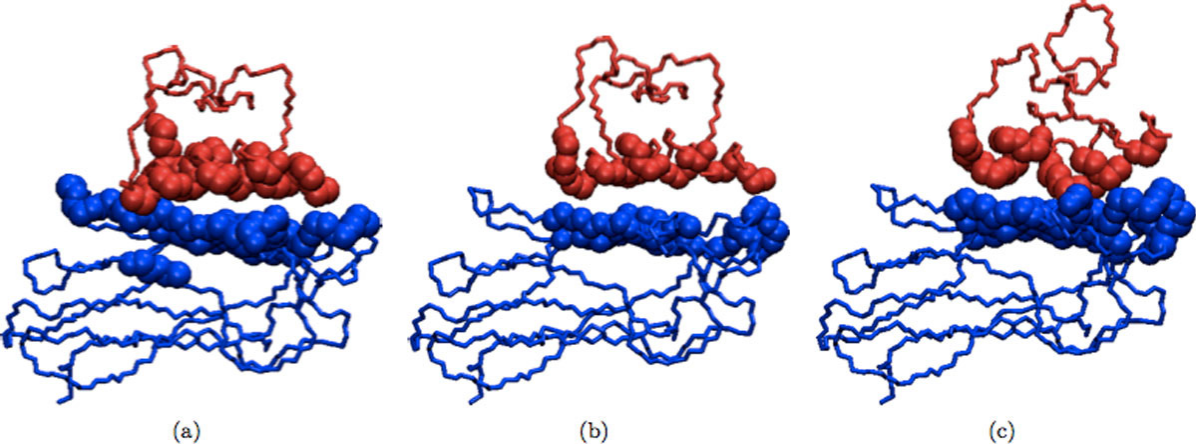
**(a) Initial docked structure (b) Refined structure (c) Native structure**. Initial docked structure for 1OHZ is shown in (a); refined version of the initial structure is shown in (b); and the native structure for 1OHZ is shown in (c). In all the following figures different chains in the protein complex are colored differently and interface atoms are drawn as spheres. Side chains and hydrogens were omitted for clarity.

**Figure 3:**
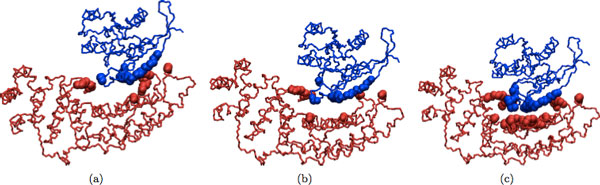
**(a) Initial docked structure (b) Refined structure (c) Native structure**. Initial docked structure for 1WQ1 is shown in (a); refined version of the initial structure is shown in (b); and the native structure is shown in (c).

**Figure 4:**
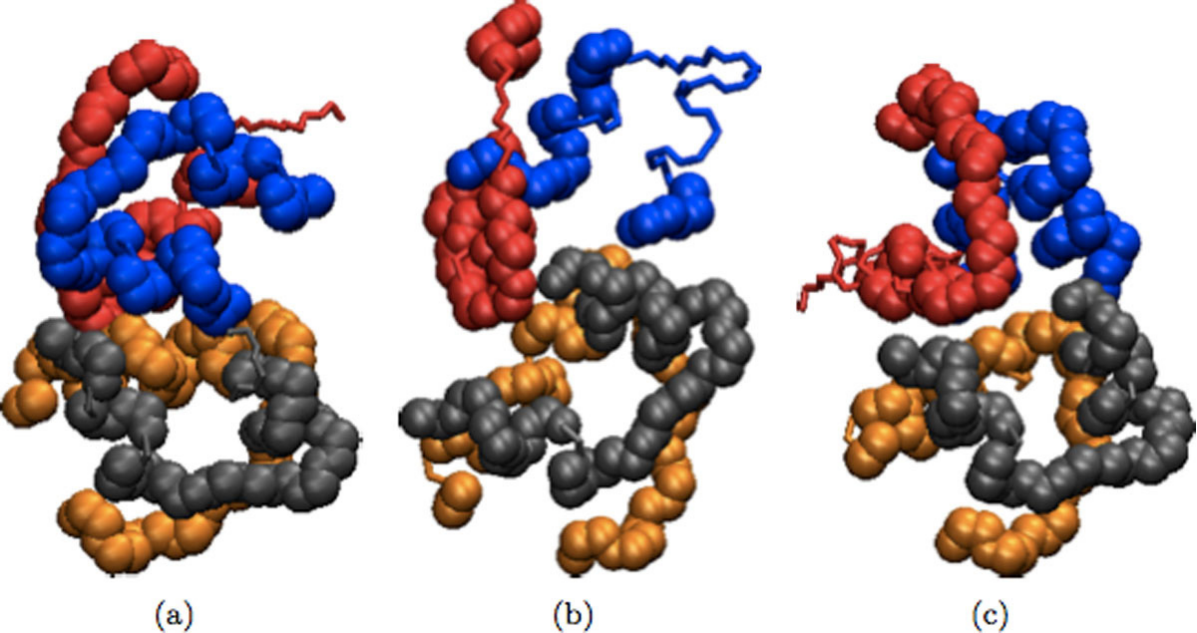
**Initial docked structure for 6RLX is shown in (a); refined version of the initial structure is shown in (b); and the native structure is shown in (c)**.

**Figure 5:**
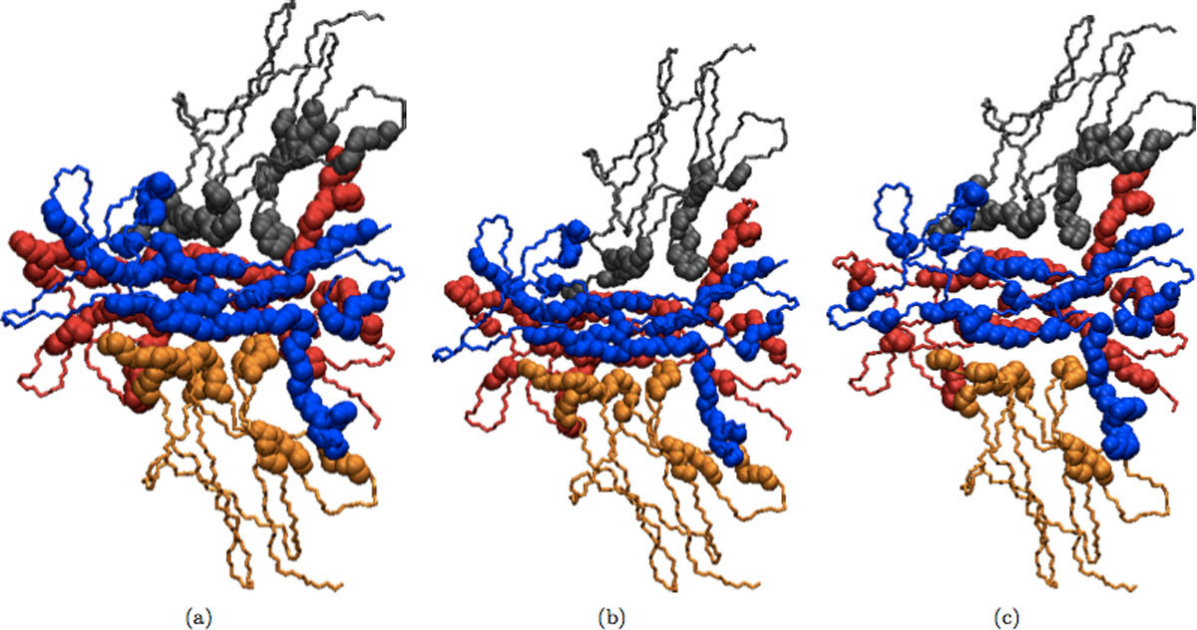
**Initial docked structure for 1WWW is shown in (a); refined version of the initial structure is shown in (b); and the native structure for 1WWW is shown in (c)**.

Refinement results of our program for dimeric and multimeric complexes are shown in Table 2 and Table 3, respectively. In addition, several examples of the docked input, refined and native structures are depicted in Figures 2, 3, 4, 5 for visual comparison. As seen, in most cases there are several structures among the top ranking complexes with better lRMSD than the input structure. In some cases, such as 1OHZ and 1WQ1, the improvement is significant - over 35%, and all resulting structures are very close to the native complex. The difference is more noticeable in the case of dimers and it can be seen in Figures 2, 3, 4, 5. In the case of multimers, even though the lRMSD difference between the input and refined structure is not big, in many cases the interface difference is rather noticeable (see for example Figure 5). Even though the organization of the input and refined structures are similar to one another and to the native structure, the interface of refined structure resembles the native structure more. This shows the potential of our method to serve as an efficient tool to improve the geometry and interface packing of coarsely docked complexes.

**Table 2:** Dimeric protein refinement results.

Protein	Input	Soln.1	Soln.2	Soln.3	Soln.4	Soln.5	Soln.6	Soln.7	Soln.8	Soln.9	Soln.10
1BDJ	4.13	3.81	3.87	3.88	3.88	3.91	3.93	3.94	3.95	3.97	4.00
1C1Y	5.45	4.84	4.94	4.94	4.97	5.03	5.06	5.06	5.13	5.16	5.18
1CSE	3.33	2.72	2.72	2.77	2.82	2.92	2.93	2.95	2.96	3.00	3.01
1DS6	4.51	4.03	4.04	4.06	4.07	4.13	4.15	4.15	4.15	4.16	4.19
1OHZ	5.05	3.38	3.52	3.72	3.81	3.96	4.06	4.23	4.41	4.41	4.61
1TX4	5.03	4.60	4.70	4.73	4.73	4.75	4.78	4.79	4.80	4.85	4.86
1WQ1	2.72	1.71	1.72	1.95	2.02	2.10	2.16	2.19	2.34	2.36	2.55

Least RMSD values in Å with respect to the native structure are shown for the initial docked structure and ten best refinement results generated by our method for each input.

**Table 3:** Multimeric protein refinement results.

Protein	Input	Soln.1	Soln.2	Soln.3	Soln.4	Soln.5	Soln.6	Soln.7	Soln.8	Soln.9	Soln.10
1I3O	3.42	3.42	3.70	3.75	3.76	3.87	3.95	4.03	4.05	4.10	4.22
1JYO	6.45	6.40	6.40	6.40	6.48	6.52	6.57	6.75	6.77	6.80	6.98
1LOG	1.63	1.64	1.65	1.81	1.81	1.82	1.85	1.88	1.93	1.94	2.00
1QGW	3.28	2.98	3.06	3.10	3.10	3.15	3.23	3.44	3.47	3.53	3.55
1VCB	3.02	3.15	3.17	3.49	3.53	3.64	3.65	3.69	3.83	3.86	3.87
1W88	4.95	4.67	4.70	4.95	5.01	5.34	5.56	5.57	5.68	5.71	5.80
1WWW	2.73	2.24	2.30	2.36	2.44	2.50	2.51	2.63	2.70	2.72	2.73
2BBK	2.07	2.07	2.09	2.27	2.41	2.52	2.59	2.73	2.74	2.84	2.87
2PRG	5.75	5.69	5.75	5.76	5.76	5.77	5.79	5.80	5.82	5.83	5.84
6RLX	6.37	5.98	6.00	6.07	6.07	6.14	6.17	6.27	6.29	6.29	6.32

Least RMSD values in Å with respect to the native structure are shown for the initial docked structure and ten best refinement results generated by our method for each input.

On the other hand, the refinement performance is not alike across different proteins. Even though our method yields better solutions than the input structure for all dimeric and some multimeric complexes, the magnitude of improvement varies from protein to protein. Indeed, there are some complexes, such as 1VCB, for which our solutions are not better than the input structure. We believe it is crucial to better understand what causes this performance difference in order to further improve our refinement method. As explained earlier, our method relies on the observation that residues on binding interfaces tend to be more conserved throughout the evolution due to their functional importance. Therefore, the conservation energy component of our scoring function is designed to favor complexes with more conserved residues on interfaces. Stated differently, structures with more clusters of conserved residues on interfaces are expected to have lower conservation energy and lower lRMSDs with respect to the native structure. On the other hand, the electrostatic energy component of the scoring function is devised to prefer complexes with lower electrostatic energy based on the assumption that native-like structures have better electrostatic interactions.

However, knowing that for some proteins, refinement results are not as good as the input structure, we performed an in-depth correlation analysis of the different scoring function components and the lRMSD to the native structure to assess our performace. For this purpose we define *ICAR *as the ratio of conserved atoms on interfaces to the total interface size. We measured the following magnitudes: (a) the ratio of conserved atoms on interfaces (ICAR) vs. lRMSD; (b) *E_TC _*vs. lRMSD; (c) ICAR vs. *E_TC _*; and (d) *E_TE _*vs. lRMSD. Ideally, ICAR would have strong negative correlation with lRMSD and *E_TC _*(a complex with more conserved atoms on the interface should have lower conservation score, be more native-like and thus have lower lRMSD with respect to the native structure), while *E_TC _*and *E_TE _*would both have strong positive correlation with lRMSD, since near-native complexes are assumed to have lower energy. To perform this correlation analysis, we generated 2000 random conformations for each docked input structure and investigated how each of these magnitudes changed with respect to one another. To calculate ICAR, we assumed a residue is conserved if its ET coverage value is lower than the following threshold, where *µ *is the mean of ET coverage values of residues in the chain, and *σ *is the standard deviation of ET coverage values of residues in the chain.

(4) threshold=μ-σ*0.5

The results of the correlation analysis are summarized in Table 4. Several points in particular are worth highlighting. First of all, ICAR vs. *E_TC _*correlation is almost always negative (except for 1BDJ and 2PRG). This confirms that our conservation scoring function correctly favors structures with more clusters of conserved atoms on interfaces as intended.

**Table 4:** Correlation coefficients for the ratio of conserved atoms on interfaces (ICAR) vs.lRMSD, total conservation energy (*E_TC_*) vs. lRMSD, total electrostatic energy (*E_TE_*) vs. lRMSD, and ICAR vs. *E_TC_*.

Protein	ICAR vs. lRMSD	*E_TC _*vs. lRMSD	*E_TE _*vs. lRMSD	ICAR vs. *E_TC_*
1BDJ	0.32	0.21	0.03	0.61
1C1Y	-0.73	0.71	0.14	-0.96
1CSE	-0.50	0.94	0.09	-0.38
1DS6	-0.63	0.62	-0.33	-0.93
1OHZ	0.39	0.07	0.61	-0.51
1TX4	-0.94	0.96	0.20	-0.99
1WQ1	0.63	-0.54	0.50	-0.86
1I3O	0.34	0.27	0.20	-0.21
1JYO	0.05	-0.43	0.12	-0.35
1LOG	0.73	-0.55	0.54	-0.69
1QGW	0.63	-0.54	0.50	-0.86
1VCB	0.33	-0.17	0.47	-0.72
1W88	-0.22	0.05	0.38	0.41
1WWW	-0.48	0.30	0.17	-0.31
2BBK	0.41	-0.07	0.49	-0.01
2PRG	0.38	-0.02	0.14	0.04
6RLX	0.63	-0.12	0.35	-0.29

Secondly, ICAR exhibits a strongly negative correlation with lRMSD correlation in most, but not all cases.

This suggests that there are cases, such as 1LOG and 6RLX, where structures with a large proportion of conserved interface atoms are less native-like, contrary to our underlying hypothesis. Whenever ICAR vs. lRMSD correlation is strong negative (e.g. 1C1Y and 1TX4), *E_TC _*shows a strong positive correlation with lRMSD as expected. In other words, structures that are closer to the native have lower conservation energy. On the other hand, when ICAR vs lRMSD is not a strong negative correlation, the conservation score is not able to favor low lRMSD structures, again as expected.

Lastly, there are certain cases where *E_TC _*does not show a positive correlation with lRMSD (e.g. 1WQ1 and 6RLX) but we are able to obtain better lRMSD structures. This is due to the positive *E_TE _*vs lRMSD correlation in these cases. This is the reason we intentionally did not mix *E_TC _*and *E_TE _*into a single energy function as also explained in our previous work [27]. The results in this paper reaffirms that observation, which suggests that we may be able to group input structures into one of two categories and employ a scoring function (*E_TC _*or *E_TE_*) selectively. This is the subject of ongoing research.

For input structures like 1LOG and 2BBK, we could not select better lRMSD structures even though *E_TC _*or *E_TE _*had relatively strong correlation with lRMSD. Analyzing them further uncovers that out of 2000 through small-scale random conformations produced for 2BBK only 7 had lower or same lRMSD as the input. In fact, our scoring function was able to select one of them. Similarly, out of 2000 random conformations produced for 1LOG only 9 had lower or same lRMSD as the input. Hence, this is either a statistical matter or generation of random conformations could have been improved to address this issue (possibly by taking symmetry that exists in some protein complexes into account), which can be considered in future work.

## Conclusions

Proteins interact to create complexes as part of their cellular function. Modeling the structure of these complexes is highly important in order to understand these processes. Here we present a refinement and re-ranking algorithm to improve the structures of coarsely docked multimeric complexes. Many protein complexes contain more than two monomers, but the vast majority of docking and refinement algorithms can only handle dimers due to the increased computational cost which causes a potential exponential increase in the runtime. Our method uses a geometry-based local search and a scoring function that is based on evolutionary conservation and pairwise interactions, relying on the observation that amino acids on binding interfaces tend to be highly conserved due to their important role. This scoring function allows us to bias our refinement scheme towards potential functional interfaces, reducing the large search space and improving the geometry and energy of the input structures. We introduced a probabilistic search scheme that allows us to escape local energy minima and enhance the diversity of selected structures. Future work includes testing our method on a larger dataset and incorporate backbone and sidechain flexibility into the search. Additionally, we plan to further investigate the difference between complexes which give better conservation score and complexes with better electrostatic energy, in order to establish an automated way to distinguish between them during the refinement process. Finally, we aim to incorporate the refinement method in a larger framework which also includes docking of multimeric complexes given only monomeric structures.

## Competing interests

The authors declare that they have no competing interests.

## Authors' contributions

B. Akbal-Delibas conducted the research. N. Haspel supervised the research. Both authors co-wrote the paper.

## Declarations

The publication costs for this article were funded by the corresponding author.

This article has been published as part of *BMC Structural Biology *Volume 13 Supplement 1, 2013: Selected articles from the Computational Structural Bioinformatics Workshop 2012. The full contents of the supplement are available online at http://www.biomedcentral.com/bmcstructbiol/supplements/13/S1.
